# Population structure and spatial distribution of *Mycobacterium tuberculosis* complex in Catalonia

**DOI:** 10.3389/fmicb.2026.1787894

**Published:** 2026-03-20

**Authors:** Antoni E. Bordoy, Vadim Leonov, Mariana G. López, Elisabet Sicart-Torres, Laia Soler, Adrián Antuori, David Panisello Yagüe, Miguel Moreno-Molina, Laura Gavaldà, Jacobo Mendioroz, Pere-Joan Cardona, Iñaki Comas, Verónica Saludes, Elisa Martró, TB-SEQ Study Group, S. González-Gómez, G. Clarà, A. C. Pelegrin, M. Torres-Puente, P. Ciruela, M. Bach, P. Gomà, P. Bach, M. Carol, P. Cano, L. Clotet, A. Despuig, L. Curto, J. Ferràs, G. Ferrús, R.M. Vileu, N. Follia, M. Sabater, E. Plasencia, M. Bosch, T. Pérez-Porcuna, À. Tarrés, M. López, H. Martínez, R. Prieto, J. P. Millet, C. Rius, S. Esteban-Cucó, E. Vicente, G. Tudó, J. González, M. T. Tórtola, T. Soler, M. D. Guerrero, I. Prats, F. Alcaide, L. Fernández, E. Cuchí, M. Garrigó, P. Costa, A. Casabella, Á. Pulido, E. Picó-Plana, J. López, G. Trujillo, N. Torrellas, X. Casas, P. Godoy, Á. Domínguez, E. Muntada, E. López-Corbeto, J. Casabona

**Affiliations:** 1 Microbiology Department, Laboratori Clínic Metropolitana Nord, Germans Trias i Pujol Research Institute and Hospital (IGTP), Badalona, Spain; 2 CIBER in Epidemiology and Public Health (CIBERESP), Instituto de Salud Carlos III, Madrid, Spain; 3 Tuberculosis Genomics Unit, Instituto de Biomedicina de Valencia (IBV, CSIC), Valencia, Spain; 4 Departament de Salut, Agència de Salut Pública de Catalunya, Generalitat de Catalunya, Barcelona, Spain; 5 Department of Genetics and Microbiology, Faculty of Biosciences, Autonomous University of Barcelona (UAB), Bellaterra, Spain; 6 CIBER in Respiratory Diseases (CIBERES), Instituto de Salud Carlos III, Madrid, Spain

**Keywords:** extrapulmonary form, hotspots, migration, spatiotemporal clusters, whole-genome sequencing

## Abstract

**Background:**

Despite being a low-incidence area, Catalonia experiences substantial migration flows from countries with high tuberculosis (TB) burden, potentially making it a focal point for various *Mycobacterium tuberculosis* complex (MTBC) lineages.

**Aim:**

We aimed to characterize the spatial structure of MTBC (sub)lineages in Catalonia, assess their associations with clinical and epidemiological factors, and identify spatiotemporal hotspots of cases belonging to the same sublineage.

**Methods:**

Whole-genome sequencing (WGS) was performed on 783 MTBC isolates collected across Catalonia from December 2021 to June 2023. Lineages and sublineages were assigned using SNP-based typing. Associations with clinical presentation, country of origin, and migration timing were evaluated using logistic regression and Fisher’s exact test. SaTScan was used to identify space–time clusters of selected (sub)lineages.

**Results:**

Lineage 4/Euro-American predominated (83%), with L4.1.2/Haarlem, L4.3/LAM, and L4.10/PGG3 widely distributed, particularly among Spanish-born individuals and long-term migrants. L1/EAI, L2/Beijing, L3/CAS, and L4.1.1/X showed geographically restricted patterns, predominantly linked to migrants from India, Pakistan, China, and Peru. L1/EAI, L3/CAS, and L4.10/PGG3 were significantly associated with extrapulmonary TB. Sublineage diversity increased from sparsely to densely populated areas. Spatiotemporal analysis identified four significant clusters involving L1/EAI, L3/CAS, and L4.10/PGG3 across seven counties, mainly within the Barcelona Metropolitan Area, with L4.10/PGG3 showing the strongest clustering.

**Conclusion:**

L4 sublineages predominate in Catalonia, whereas L1/EAI, L2/Beijing, and L3/CAS are more geographically restricted and predominantly associated with migrants. The presence of sublineage-specific hotspots in dense urban areas highlights the role of migration dynamics and urbanization in shaping TB transmission.

## 1 Introduction

Tuberculosis (TB) remains a major global health challenge, exacerbated by limited vaccine efficacy, comorbidities such as HIV, and diversity of circulating TB strains (Martini et al., 2020; World Health Organization, 2023a). Addressing these challenges requires an enhanced understanding of TB epidemiology and the genetic variability of its causative agent, the *Mycobacterium tuberculosis* complex (MTBC).

Whole genome sequencing (WGS) has identified nine main MTBC lineages with varied geographic distributions. L1/East-African-Indian (EAI) and L3/Central-Asian (CAS) are predominant in Asia and East Africa, while L2/Beijing and L4/Euro-American (EA) are globally distributed (Wiens et al., 2018; Coscolla et al., 2021). L5, L6, L7, L8, and L10 are confined to Africa (Gagneux, 2012; Menardo et al., 2021; Getahun et al., 2024; Guyeux et al., 2024). Contemporary intense migration flows from high-burden countries make the population structures of the MTBC lineages vary uniquely across countries and can result in distinct distributions even within a single country (Getahun et al., 2024; Xu et al., 2022), which should be considered when developing TB control strategies.

Catalonia, which is recognized as a low TB incidence region (13.0 cases/100,000 inhabitants in 2023) (Gavaldà Mestre et al., 2023) with a high acceptance of migration flows, presents a unique demographic context where TB disproportionally affects migrants, which represented 62.4% of the diagnosed cases in 2023, with an associated incidence rate of 36.1 cases/100,000 inhabitants, compared to 6.3 cases/100,000 inhabitants in the native population (Gavaldà Mestre et al., 2023). In addition, substantial geographic variability in TB incidence also exists within Catalonia, with a range of 2.9 to 28.4 cases/100,000 inhabitants at the county level. Population-based MTBC genomic data for Catalonia, generated for the first time by the TB-SEQ project, provides a valuable resource for understanding these epidemiological differences. Initiated in late 2021 and integrated into formal epidemiological surveillance in 2022 under the Public Health Agency of Catalonia (Saludes et al., 2023), the project systematically applies centralized WGS to all culture-positive TB isolates.

In this sub-study, we examined the spatial and population structure of MTBC lineages in Catalonia (December 2021–June 2023), their associations with clinical and epidemiological variables, and the presence of spatiotemporal hotspots of cases belonging to the same sublineage. This approach aims to clarify how lineage diversity, migration dynamics, and urban density contribute to the TB burden in Catalonia and to guide the strategic deployment of future targeted public health interventions.

## 2 Materials and methods

### 2.1 Study area

Catalonia is an Autonomous Community in the northeastern part of Spain, covering an area of 32,113 km^2^, accounting for approximately 6.35% of Spain’s total area and 16.7% of its population with about 7.90 million inhabitants in 2023^1^. Administratively, Catalonia is divided into four provinces and 43 counties.

### 2.2 Population, design and data collection

In Catalonia, TB prevention and control is managed by the Public Health Surveillance and Emergency Response Service. After diagnosis, TB cases are mandatorily and individually notified by the physicians responsible for the diagnosis and treatment of the disease. Additionally, laboratories notify the corresponding microbiological data. For each notified case, public health agents from a network of nine territorial epidemiological surveillance services distributed across the territory perform contact-tracing investigations, which is the gold standard procedure to detect epidemiological outbreaks. The territorial epidemiological surveillance services fill in an epidemiological questionnaire, and this information is stored in the Epidemiological Register of Catalonia (REC). The registry, which includes socio-demographic (including country of origin and timing of immigration), clinical (including form of TB), and microbiological data (including smear microscopy results and type of specimen), as well as risk factors for TB disease, was used to obtain relevant information.

In the TB-SEQ population-based genomic surveillance project (from December 1, 2021, to June 30, 2023), every culture-confirmed case of MTBC, whether pulmonary or extrapulmonary, was sequenced and genotyped for patients residing in Catalonia. These cultures were obtained from clinical samples collected by a network of 46 sites, including public and private hospitals and laboratories, primary care centers, prisons, and a clinic specialized in TB treatment, representing microbiologically notified TB cases from all 43 counties. Grown cultures were shipped for centralized WGS at the coordinator laboratory. Results were shared with public health officers, who unified genomic and epidemiological information.

### 2.3 Genome sequencing and bioinformatic analysis

DNA was extracted using the EZ-1 DSP Viral Kit (Qiagen), libraries were prepared with Nextera XT or Illumina DNA Prep kits, and sequencing was performed on Illumina MiSeq or NextSeq 1,000 platforms. Paired-end reads were quality-trimmed, filtered with KRAKEN (Wood and Salzberg, 2014), and processed through a standardized TB bioinformatic pipeline (Comas et al., 2013; Chiner-Oms et al., 2019; Cancino-Muñoz et al., 2019; Meehan et al., 2019) and maximum-likelihood phylogenetic reconstruction in IQ-TREE v1.6.12. Lineages were assigned using established phylogenetic SNP markers and manually curated for monophyly. (Sub)lineage nomenclature followed that proposed by Coll et al. (2014). The phylogenetic tree is provided in Supplementary Figure S1. Drug resistance prediction was performed comparing single-nucleotide polymorphism and indel data with the WHO catalogue of drug-resistant associated mutations in the *M. tuberculosis complex* (World Health Organization, 2023b).

For downstream analyses, lineages or sublineages with at least 20 cases were considered. The included groups were: L1 (East-African-Indian, EAI), L2 (Beijing), L3 (Central-Asian, CAS), L4.1.1 (X), L4.1.2 (Haarlem), L4.3 (Latin-American-Mediterranean, LAM), and L4.10 (Principal Genetic Group 3, PGG3). Lineages with fewer than 20 isolates (hereafter referred to as minor (sub)lineages) — including L4.1.3, L4.2, L4.5, L4.6, L5 (Manu1), L6 (Manu2), *Mycobacterium bovis*, *M. bovis* BCG, and undefined L4 strains — were excluded.

In the following text, the terms lineage/sublineages, and spatiotemporal cluster/hotspots are used interchangeably where appropriate and not critical.

### 2.4 Statistical analysis

Uni- and multivariate logistic regression with Firth’s penalized likelihood was applied to assess the association between the most frequent MTBC (sub)lineages and positive microscopy and form of TB. The L4.1.2/Haarlem was selected as the reference category because of its relatively high frequency and broad geographical distribution across Catalonia. Firth’s penalized likelihood was chosen to address small sample sizes and potential separation in the data. Predictor variables examined included age, sex, and immigration timing (recent immigrant, long-term immigrant, Spanish-born). Immigration timing was categorized into recent migrants (≤2 years) and long-term migrants (>2 years) using self-reported year of arrival. Adjusted odds ratios (aORs) and 95% confidence intervals (CIs) were reported with significance defined as *p* < 0.05.

Fisher’s exact test was used to examine the relationship between immigration timing and MTBC (sub)lineages. Association between (sub)lineages and country of origin were evaluated using Firth’s logistic regression following a global Fisher’s exact test (or Chi-square test when *n* > 50) that identified significant differences. ORs, 95% CIs, and *p*-values were reported, with *p*-values adjusted for multiple testing using the false discovery rate (FDR) method; associations were considered statistically significant at FDR-adjusted *p* < 0.05. Spain was used as the reference category for the variable country of origin. For immigration timing, Spanish-born individuals served as the reference group against which recent and long-term migrants were compared.

(Sub)lineage proportional abundance per county was used to calculate Shannon and Simpson indices. Counties were conditionally classified as densely populated (≥1,500 inhabitants/km^2^), moderately populated (150–1,500 inhabitants/km^2^) and sparsely populated (<150 inhabitants/km^2^) according to publicly available data (see text footnote 1). Differences in (sub)lineage diversity indices were assessed using Kruskal-Wallis test.

The clinical and sociodemographic variables and (sub)lineage assignments used in all statistical analyses are provided in Supplementary Table S1. All analyses were conducted in R 2023.06.1 + 524.

### 2.5 Scan statistics analysis

Kulldorff’s scan statistics tool is effective in identifying areas where the observed number of cases significantly deviates from what would be expected under spatial randomness (Kulldorff, 1997). A circular window across the study area, varying in size and location, to identify clusters with higher or lower prevalence of MTBC lineages. The window with the highest likelihood ratio (LLR) was considered the primary cluster, and additional significant windows were treated as secondary clusters. Statistical significance was assessed with 9,999 Monte Carlo simulations, with *p* < 0.05 indicating a significant cluster. Relative risk (RR) was calculated for each cluster. Each MTBC (sub)lineage was analyzed separately using a spatiotemporal Poisson model with a maximum radius of 30 km to minimize overlap across Catalonia. Different spatial and temporal window sizes produced no meaningful changes in cluster characteristics. All data for spatiotemporal analysis is provided in Supplementary Table S2. SaTScan^™^ software v10.1 was used for spatiotemporal clustering analysis, and QGIS 3.28.3 software was employed to visualize the MTBC spatiotemporal clusters and lineages distribution.

## 3 Results

### 3.1 Population characteristics

Over the study period, 783 positive TB cultures underwent WGS successfully. The dataset represented 73.0% (783/1072; personal communication, L. Gavaldà) of all TB culture-positive cases notified during the same period. The included cases were 551 males (70.4%; mean age 45.5 ± 18.6 years), 231 females (29.5%; mean age 43.8 ± 21.1), and for one case sex information was not available (0.1%). Regarding national origin, 60.8% (476/783) of the patients were migrants from 47 different countries. The most frequent countries of origin were Morocco (22.9%, *n* = 109), Pakistan (12.2%, *n* = 58), and Peru (9.2%, *n* = 44). Among migrants, 90 (18.9%) were classified as recent, 349 (73.3%) as long-term migrants, and 38 (8.0%) had an unknown migration timing. 36.3% (284/783) of the cases had positive microscopy results from respiratory specimens, and 79.8% (625/783) presented with pulmonary or mixed forms of TB, while 20.2% (158/783) had exclusively extrapulmonary TB (EPTB).

### 3.2 MTBC population structure

Seven MTBC lineages were identified (Supplementary Figure S1). Among them, L4 accounted for 83.0% (*n* = 650) of the isolates, followed by L3 (*n* = 54, 6.9%), L2 (*n* = 27, 3.4%), L1 (*n* = 20, 2.6%), *M. bovis* (*n* = 14, 17.9%), L6 (*n* = 11, 1.4%), and L5 (*n* = 3, 0.4%). An undefined L4 sublineage (*n* = 4, 0.5%) was also identified.

**Figure 1:**
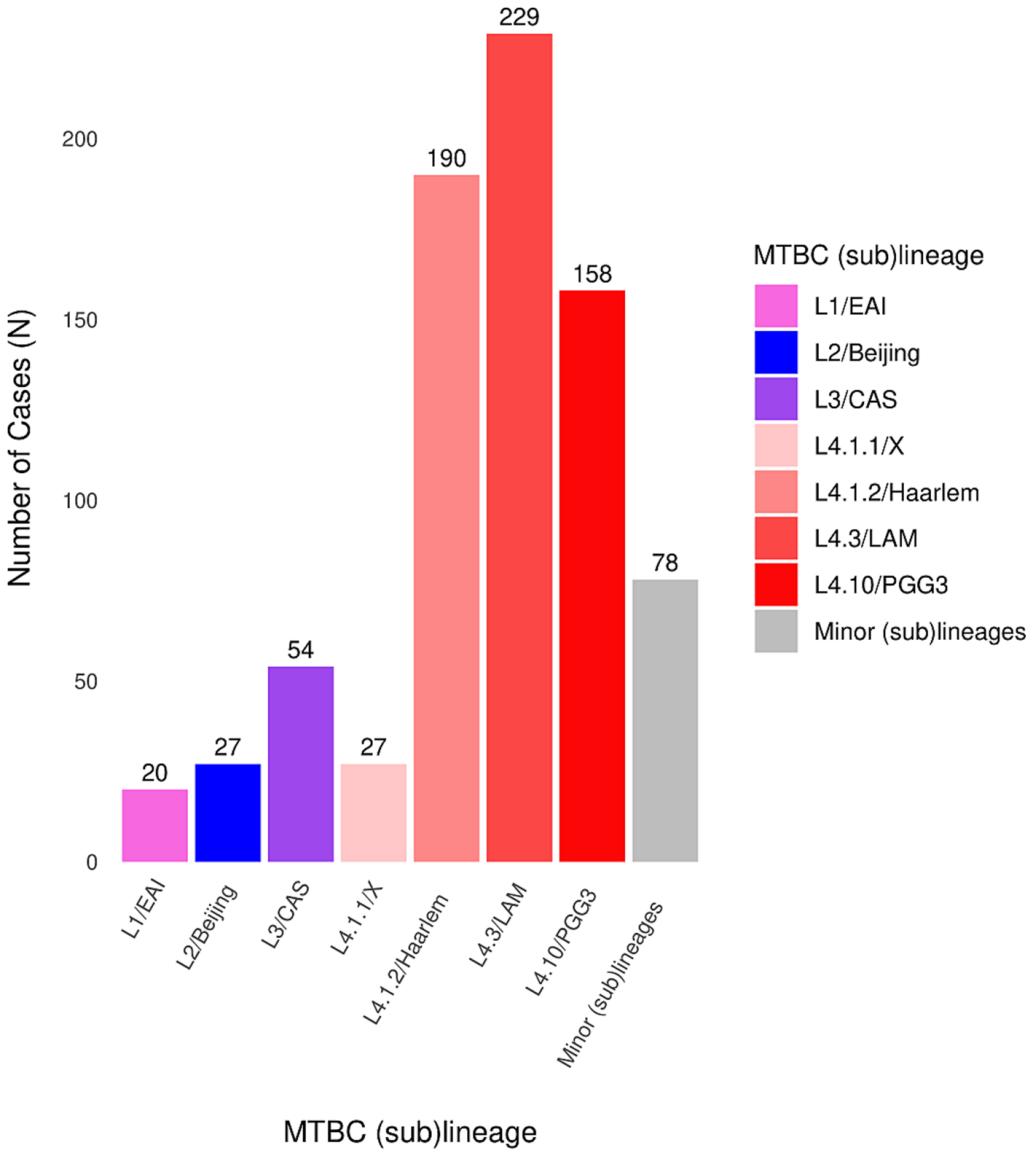
Distribution of MTBC (sub)lineages among culture-positive TB cases in Catalonia from December 2021 to June 2023 (*N* = 783).

The frequency distribution of circulating (sub)lineages is shown in Figure 1. Within L4, three sublineages dominated the population: L4.3/LAM (29.2% of all isolates), L4.1.2/Haarlem (24.3%), and L4.10/PGG3 (20.2%). Together, they accounted for 73.7% of L4 cases. Minor MTBC (sub)lineages are displayed collectively in Figure 1.

Overall, according to genomic resistance prediction, most isolates were susceptible (*n* = 729, 93.1%) to isoniazid, rifampicin, ethambutol, and pyrazinamide. 46 (5.9%) cases were monoresistant, including 26 (3.3%) monoresistant to isoniazid, 16 (2.0%) monoresistant to pyrazinamide, three (0.4%) monoresistant to rifampicin, and one (0.1%) monoresistant to ethambutol. Besides, 5 (0.6%) cases were monoresistant to fluoroquinolones. There were seven (0.9%) multidrugresistant tuberculosis cases and a single (0.1%) pre-extensively drug-resistant tuberculosis case.

### 3.3 Geographic distribution of (sub)lineages

Most culture-confirmed TB cases occurred in the Barcelonès county (*n* = 261), followed by its surrounding counties: Vallès Occidental (*n* = 86), Baix Llobregat (*n* = 60), and Maresme (*n* = 50), reflecting a concentration of cases within the Barcelona Metropolitan Area. In contrast, counties such as Ribera d’Ebre, Cerdanya, Conca de Barberà, Garrigues, Pallars Jussà, Segarra, and Solsonès reported only 1–2 cases each. Thirteen counties had no reported cases. The remaining 19 counties reported between 3 and 49 cases each.

**Figure 2:**
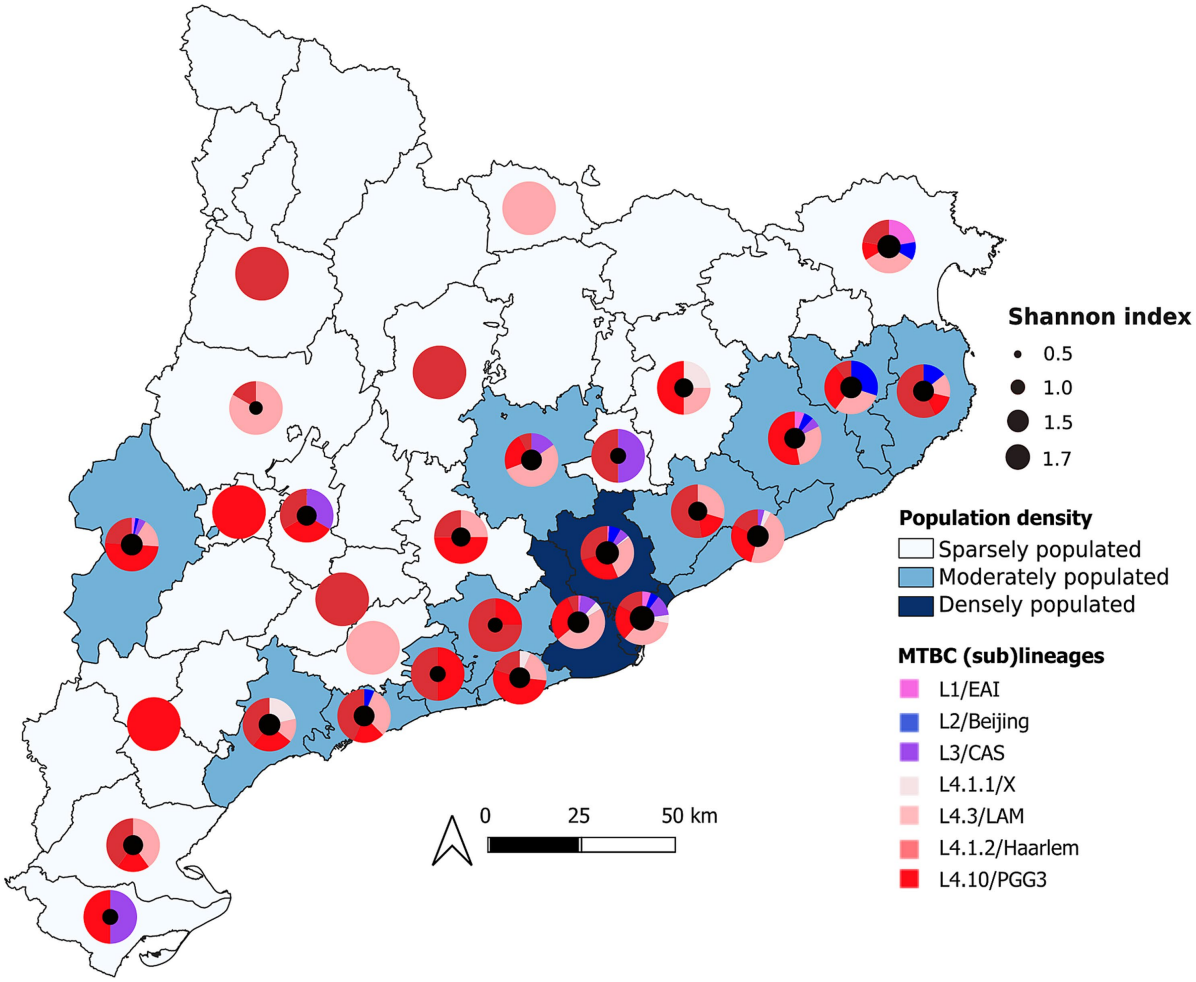
Spatial distribution and regional diversity of major MTBC (sub)lineages in Catalonia. Pie charts depict the proportional representation of 705 MTBC isolates across Catalonian counties, colored by (sub)lineages. The choropleth layer illustrates population density.

L4/EA predominated across Catalonia and accounted for 84.7% (39/46) of cases in sparsely populated areas (Figure 2). Within the Barcelona Metropolitan Area, the dominant sublineages were L4.3/LAM (40.0%, *n* = 163), L4.1.2/Haarlem (24.5%, *n* = 112), and L4.10/PGG3 (17.7%, *n* = 81), which were also the most frequently detected sublineages in surrounding counties. In contrast, L1/EAI, L2/Beijing, and L3/CAS were largely concentrated in Barcelonès and Vallès Occidental, with limited and sporadic distribution in other counties (29 cases across 9 counties).

Visual inspection of the geographic distribution map (Figure 2) revealed that more peripheral counties exhibit lower (sub)lineage diversity. Shannon diversity differed significantly across density groups (Kruskal-Wallis χ^2^ = 22.68, df = 2, *p* < 0.001), being higher in densely populated counties than in moderately (*p* = 0.0001) or sparsely populated counties (*p* < 0.001), with no significant difference between moderately and sparsely populated counties (*p* = 0.316). In contrast, Simpson diversity showed no significant difference across density groups (Kruskal-Wallis χ^2^ = 1.24, d = 2, *p* = 0.54), indicating stable dominance patterns along the sparsely to densely populated gradient.

### 3.4 Disease and sociodemographic factors associated with dominant (sub)lineages

We assessed the association between MTBC (sub)lineage and two variables: microscopy results (positive vs. negative) and clinical form of TB (extrapulmonary vs. pulmonary/mixed) (Table 1).

**Table 1:** Multivariate analysis between TB-related characteristics and major MTBC (sub)lineages.

Variables	Disease-related characteristics					
	Positive microscopy			EPTB		
	n/N	aOR [95% CI]	*p*-value	n/N	aOR [95% CI]	*p*-value
L1/EAI	5/6	3.02 [0.57, 30.49]	0.207	8/20	3.73 [1.33, 10.2]	**0.013**
L2/Beijing	11/20	0.68 [0.26, 1.78]	0.423	3/27	0.92 [0.23, 2.83]	0.900
L3/CAS	11/22	0.58 [0.23, 1.45]	0.240	21/54	3.81 [1.85, 7.86]	**<0.001**
L4.1.1/X	9/17	0.72 [0.26, 1.98]	0.515	2/27	0.60 [0.11, 2.07]	0.451
L4.1.2/Haarlem	88/143	Ref	Ref	25/190	Ref	Ref
L4.3/LAM	83/155	0.73 [0.45, 1.16]	0.178	42/229	1.36 [0.79, 2.36]	0.269
L4.10/PGG3	59/98	0.94 [0.55, 1.61]	0.832	34/158	1.89 [1.06, 3.39]	**0.029**
Age	-	0.98 [0.97, 0.99]	**<0.001**	-	1.01 [1.00, 1.02]	**0.038**
Sex (male)	205/344	1.38 [0.90, 2.12]	0.137	76/492	0.43 [0.29, 0.64]	**<0.001**
Immigration timing (recent)	35/56	0.91 [0.47, 1.77]	0.775	10/80	0.97 [0.42, 2.12]	0.941
Immigration timing (long-term)	115/206	0.70 [0.45, 1.09]	0.112	83/338	1.97 [1.23, 3.17]	**0.004**

n/N, count of cases with the feature per total in (sub)lineage; EPTB, extrapulmonary tuberculosis; aOR, adjusted odds ratio; CI, confidence interval. Bold *p*-values indicate statistical significance.

The proportion of microscopy-positive cases did not differ substantially across (sub)lineages, indicating no consistent association between lineage and smear positivity. Age was the only independent predictor, with a 0.98-fold decrease in odds of smear positivity per year of age (2%, *p* < 0.001). Adjustment for sex and immigration timing did not substantially change the results.

EPTB was observed in 8/20 (40.0%) patients infected with L1/EAI, 21/54 (38.9%) with L3/CAS, and 34/158 (21.5%) with L4.10/PGG3, compared with 25/190 (13.2%) among those infected with L4.1.2/Haarlem. Accordingly, EPTB was 3.7-, 3.8-, and 1.9-fold more likely in patients infected with L1/EAI, L3/CAS, and L4.10/PGG3, respectively, compared with L4.1.2/Haarlem. Males were 0.4-fold less likely to present with EPTB compared with females. Increasing age was associated with a 1.0-fold higher likelihood of EPTB per year. Long-term immigrants were 2.0-fold more likely to develop EPTB compared with Spanish-born individuals, while recent immigrants showed no significant difference. L1/EAI occurred exclusively in migrants (95% long-term). L2/Beijing was 7.8- and 3.3-fold more frequent in recent and long-term migrants, respectively, compared with Spanish-born patients. L3/CAS was 22.5- and ~20-fold higher in recent and long-term migrants. L4.1.1/X and L4.3/LAM showed no difference by migration, L4.1.2/Haarlem was slightly less frequent in migrants, and L4.10/PGG3 was 4.5- and 1.9-fold more common in Spanish-born than in recent and long-term migrants (Table 2).

**Table 2:** Comparative analysis of migration characteristics of main MTBC (sub)lineages.

MTBC (sub)lineage	Immigration status						
	Spanish-born patients*	Recent migrants			Long-term migrants		
	*n*	*n*	OR [95% CI]	*p*-value	*n*	OR [95% CI]	*p*-value
L1/EAI (*N* = 20)	0	1	NA	NA	19	NA	NA
L2/Beijing (*N* = 27)	4	8	7.80 [2.02, 36.4]	**<0.001**	15	3.28 [1.03, 13.7]	**0.034**
L3/CAS (*N* = 54)	2	11	22.5 [4.74, 212.5]	**<0.001**	41	19.6 [5.01, 168.7]	**<0.001**
L4.1.1/X (*N* = 27)	9	5	2.05 [0.52, 7.07]	0.197	13	1.24 [0.48, 3.33]	0.670
L4.1.2/Haarlem (*N* = 190)	94	18	0.60 [0.31, 1.09]	0.099	78	0.62 [0.43, 0.89]	**0.009**
L4.3/LAM (*N* = 229)	89	30	1.31 [0.75, 2.26]	0.346	110	1.04 [0.73, 1.48]	0.863
L4.10/PGG3 (*N* = 158)	88	7	0.22 [0.08, 0.50]	**<0.001**	63	0.52 [0.35, 0.76]	**<0.001**

*, reference group; *N*, total number; *n*, cases per subgroup; OR, odds ratio; CI, confidence interval. Bold *p*-values indicate statistical significance.

Significant associations were observed between country of origin and specific MTBC (sub)lineages (Table 3). The L1/EAI lineage was almost exclusively detected among South Asian long-term migrants, with no cases in Spanish-born patients. For L2/Beijing, significant associations were observed in migrants from China, India, and Peru (OR = 28.7, 6.55, and 4.43, respectively), with two-thirds being long-term residents. L3/CAS was concentrated in South Asian long-term migrants (79.6%, *n* = 39), with strong associations for India (OR = 12.4) and Pakistan (OR = 110). L4.1.1/X was linked to Peruvian migrants (OR = 5.6). L4.1.2/Haarlem sublineage was significantly less common among migrants from Senegal, with 4.3-fold lower odds than Spanish-born individuals. L4.3/LAM was more frequent among individuals from Morocco and Senegalese (OR = 1.95 and 2.83, respectively) but markedly underrepresented in those from Pakistan (OR = 0.06). Lastly, the L4.10/PGG3 sublineage was notably less frequent in Peruvian individuals, with OR = 0.31, corresponding to ~70% lower odds relative to Spanish-born individuals.

**Table 3:** Significant associations of MTBC (sub)lineage with country of origin (post-hoc Firth regression analysis).

Country	MTBC (sub)lineage	*n*	OR [95% CI]	p_adj_-value
Pakistan (*N* = 51)	L1/EAI	6	6.31 [2.23, 16.1]	**0.009**
	L3/CAS	38	110 [51.6, 252]	**<0.001**
	L4.3/LAM	1	0.06 [0.01–0.21]	**<0.001**
India (*N* = 17)	L1/EAI	3	9.26 [2.25. 30.0]	**0.022**
	L2/Beijing	3	6.55 [1.62, 20.6]	**0.041**
	L3/CAS	8	12.4 [4.58, 32.9]	**<0.001**
China (*N* = 8)	L2/Beijing	4	28.7 [7.01, 117]	**<0.001**
Peru (*N* = 40)	L2/Beijing	5	4.43 [1.49, 11.2]	**0.037**
	L4.1.1/X	6	5.65 [2.05, 13.8]	**0.011**
	L4.10/PGG3	3	0.31 [0.08, 0.81]	**0.046**
Morocco (*N* = 98)	L4.3/LAM	45	1.95 [1.26, 3.00]	**0.016**
Senegal (*N* = 30)	L4.1.2/Haarlem	2	0.23 [0.05, 0.70]	**0.027**
	L4.3/LAM	17	2.83 [1.37, 5.96]	**0.023**

*N*, total number; *n*, cases per subgroup; OR, odds ratio; CI, confidence interval; p_adj_-value, FDR-adjusted *p*-value (Benjamini-Hochberg correction). Bold *p*-values indicate statistical significance.

### 3.5 Hotspot analysis of MTBC (sub)lineages

Two lineages and one sublineage of MTBC created significant primary spatiotemporal clusters: L1/EAI, L3/CAS, and L4.10/PGG3 (Table 4) in 7 counties of Catalonia (mainly in the Barcelona Metropolitan Area): Vallès Occidental, Barcelonès, Baix Llobregat, Maresme, Moianès, Vallès Oriental, and Baix Camp. The L2/Beijing, L4.1.1/X, L4.1.2/Haarlem, and L4.3/LAM (sub)lineages, did not form any clusters under the SaTScan settings used. The distribution of hotspot areas is presented in cluster maps (Supplementary Figure S2).

**Table 4:** Retrospective spatiotemporal cluster analysis of MTBC (sub)lineages.

MTBC (sub)lineage	Location, size and time	RR	LLR	*p*-value	Proportional composition cluster vs. lineage (Spanish-born patients, recent migrants, long-term migrants)
L1/EAI^b^^,^^c^ (*N* = 9)	Barcelonès 0 km 2021/12/1 to 2022/6/30	7.22	7.98	**0.008**	(0, 0, 1.0) vs. (0, 0.05, 0.95)
L3/CAS^b^^,^^c^ (*N* = 24)	Barcelonès, Baix Llobregat, Vallès Occidental, Moianès 26.03 km 2022/10/1 to 2023/6/30	3.54	10.26	**0.002**	(0.08, 0.12, 0.79) vs. (0.04, 0.20, 0.76)
L4.10/PGG3^a^^,^^c^ (*N* = 53, *N* = 11)	Vallès Oriental, Maresme, Moianès, Vallès Occidental 26.57 km 2023/1/1 to 2023/6/30	6.74	48.00	**<0.001**	(0.47, 0.06, 0.47) vs. (0.56, 0.04, 0.40)
	Baix Camp 0 km 2023/1/1 to 2023/3/31	19.79	22.03	**<0.001**	(0.54, 0, 0.45) vs. (0.56, 0.04, 0.40)

RR, Relative Risk; LLR, Log-Likelihood Ratio.

a Significant for Spanish-born vs. recent migrants.

b Significant for Spanish-born vs. long-term migrants.

c Significant for long-term vs. recent migrants (Fisher’s exact or Chi-square test). Bold *p*-values indicate statistical significance.

L1/EAI and L3/CAS clusters involved predominantly long-term migrants, while L4.10/PGG3 clusters included mainly Spanish-born and long-term migrants. The most extensive clustering was observed for L4.10/PGG3, which formed a primary cluster in Vallès Oriental, Maresme, Moianès, Vallès Occidental, with a secondary cluster identified in Baix Camp. The primary cluster had a long duration of 6 months, while the secondary cluster exhibited the highest RR value of 19.79, indicating a short-term period of intense case accumulation lasting 3 months in that area.

## 4 Discussion

This study analyzed the composition and spatiotemporal distribution of MTBC (sub)lineages in Catalonia identified by WGS, providing an overview of the region’s TB genetic landscape, which was dominated by L4 sublineages. Spatial mapping and statistical analyses revealed a clear gradient in MTBC lineage diversity across population density groups. Sublineage richness and evenness increased with population density. Densely populated areas, such as the Barcelona Metropolitan Area, displayed no dominant sublineages, while intermediate and sparsely populated peripheral counties such as Segrià, Garraf, Bages, and Alt Empordà showed reduced diversity and were often dominated by one or two L4 sublineages.

Regression analysis revealed that L1/EAI, L3/CAS, and L4.10/PGG3 were significantly associated with EPTB compared to L4.1.2/Haarlem. The latter two observations contrast with previous reports suggesting that L1/EAI strains were primarily responsible for this disease form (Du et al., 2023). However, our results should be interpreted cautiously due to the relatively small number of L1/EAI and L3/CAS cases, which limited subgroup analyses. Nevertheless, the pattern observed in L3/CAS aligns with earlier findings from the USA, showing a higher prevalence of EPTB irrespective of regional or racial/ethnic factors, nutritional status, or BCG vaccination history (Click et al., 2012). Additionally, origin is the strongest predictor of EPTB, with individuals from South-East Asia and the Eastern Mediterranean showing higher odds than Europeans, suggesting a role of genetic susceptibility or delayed case detection (Rachwal et al., 2025). Male patients were less likely to present with EPTB than females, consistent with previous epidemiological studies (Eddabra and Neffa, 2020; Pang et al., 2019). Long-term immigrants were more likely to develop extrapulmonary disease in line with findings from European surveillance data (Hayward et al., 2021). To our knowledge, this is the first study to detect the potential association of L4.10/PGG3 with EPTB, which should be confirmed in additional studies.

No significant associations were found between any lineage and positive microscopy results from respiratory specimens, suggesting that MTBC detectability is lineage-independent and likely driven by extrinsic factors such as overcrowding, housing conditions, social vulnerability, or healthcare access. Age showed modest associations with clinical presentation: older individuals exhibited lower smear positivity and slightly higher odds of EPTB. These patterns align with previous reports attributing such trends to immunosenescence and comorbidities among older adults (Padberg et al., 2015).

Migration patterns strongly influence the distribution of MTBC (sub)lineages in Catalonia. L1/EAI and L3/CAS were almost exclusively detected in migrant populations, largely from India and Pakistan, consistent with their endemicity in South Asia (Menardo et al., 2021). The absence of L1/EAI among Spanish-born individuals is consistent with importation rather than local circulation. In contrast, 80% of L3/CAS occurred in long-term migrants residing in the Barcelona Metropolitan Area, suggesting a local transmission hub in addition to ongoing importation. The L2/Beijing lineage was significantly associated with migrants from China, India, and Peru, reflecting both importation and sustained transmission within the metropolitan region.

Conversely, L4.1.2/Haarlem and L4.10/PGG3 were negatively associated with migrants from Senegal, Morocco, Pakistan, and Peru, indicating that these globally distributed sublineages primarily circulate among Spanish-born individuals and long-term migrants in Catalonia. In contrast, L4.1.1/X and L4.3/LAM, displayed significant positive associations with specific migrant groups, including Peruvian (L4.1.1/X) and Moroccan or Senegalese (L4.3/LAM) individuals.

Identified patterns for MTBC (sub)lineages in Catalonia align with global trends, where generalist L4 sublineages such as L4.1.2/Haarlem, L4.3/LAM, and L4.10/PGG3 are prevalent worldwide, whereas L1/EAI, L2/Beijing, and L3/CAS remain more geographically restricted to South Asia, China, and East Africa (Menardo et al., 2021; Stucki et al., 2016).

Overall, we identified seven counties with four spatiotemporal clusters in Catalonia, six of them forming a continuous geographical area that acts as a hotspot for three MTBC (sub)lineages. In the case of L3/CAS, which is not endemic to the European continent; it is important to note that the hotspot comprising Barcelonès, Baix Llobregat, Vallès Occidental and Moianès is significantly populated by migrants from Pakistan and India, suggesting that importation predominates over local transmission as L3/CAS was rarely identified in other nationalities. Contrary to expectations, no significant hotspots were detected for the L4.1.2/Haarlem or L4.3/LAM sublineages. Their widespread but relatively even distribution across Catalonia suggests an absence of spatial clustering, consistent with transmission largely occurring among Spanish-born individuals and long-term migrants rather than recent migrants. L4.10/PGG3 demonstrated an outstanding clustering pattern with the highest spatial significance and RR values, especially in Vallès Oriental, Maresme, Moianès, Vallès Occidental, and Baix Camp. The cluster in Baix Camp is notably small and short-lived. These clusters were composed mainly of Spanish-born individuals and long-term migrants, indicating sustained local transmission. Importantly, most spatiotemporal clusters were centered in the Barcelona Metropolitan Area, which is characterized by intense industrial activity, high population density, and immigration rates. Although research on the spatial epidemiology of TB in Spain is limited, Gómez-Barroso et al. (2013) previously identified the Barcelona Metropolitan Area as the most likely TB cluster in Spain based on cumulative reported cases.

The presence of sublineage-specific hotspots in the Barcelona Metropolitan Area underscores the contributions of migration dynamics and urban density to TB transmission. Nevertheless, our results suggest that transmission dynamics in Catalonia differ between globally distributed and geographically restricted sublineages. Overall, these findings align with the reported value of integrating genomic, spatial, and epidemiological approaches to support targeted TB control strategies (Lan et al., 2025). Ongoing genomic clustering analyses of WGS data will further clarify local transmission dynamics, distinguish between imported and locally transmitted cases, and validate the epidemiological patterns described here. The integration of genomic data with spatiotemporal surveillance is being implemented in Catalonia to enhance TB control efforts by enabling more precise targeting of high-risk populations and locations (Pérez-Porcuna et al., 2025).

Several factors should be considered when interpreting our findings. Firstly, the dataset represents 73% of all culture-positive TB cases notified during the study period (783/1072), with potential selection bias implications if sequenced and non-sequenced cases differed systematically. A higher proportion of sequenced isolates could have led to detecting larger or additional spatiotemporal clusters. However, we believe that the data available was representative of the geographic patterns in Catalonia. Secondly, we used county-level aggregated data, which may have reduced spatial resolution. Thirdly, sparse populations in some areas limited spatial analyses; to address this, we applied a Poisson model without restricting the minimum number of cases for detecting high-rate clusters. Finally, due to the small sample sizes for L1/EAI, L2/Beijing, and L4.1.1/X, we applied Firth logistic regression to reduce bias, with results consistent with standard logistic models. The consistency of results between Firth and standard logistic models supports the robustness of our findings.

While ongoing genomic clustering and epidemiological analyses are expected to deepen our understanding of MTBC transmission in Catalonia, this study demonstrates that integrating genotyping and geospatial statistics enhances understanding of local dynamics and supports further monitoring of transmission. Here, we provide a detailed epidemiological portrait of MTBC (sub)lineages circulating in Catalonia between December 2021 and June 2023, highlighting the complex interplay between migration, urban density, and (sub)lineage distribution. The predominance of generalist L4 sublineages across the region contrasts with the geographically restricted and migrant-associated distribution of L1/EAI, L2/Beijing, and L3/CAS lineages. Our integration of space–time cluster analysis highlighted the Barcelona Metropolitan Area as a key location of lineage-specific hotspots.

## Data Availability

The datasets presented in this study can be found in online repositories. The names of the repository/repositories and accession number(s) can be found in the article/Supplementary material.

## Footnotes

1
https://www.idescat.cat/

## Group member of TB-SEQ Study Group

Germans Trias i Pujol Research Institute and Hospital (IGTP): S. González-Gómez, G. Clarà, A. C. Pelegrin. IBV-CSIC: M. Torres-Puente. Public Health Agency of Catalonia: P. Ciruela, M. Bach, P. Gomà, P. Bach, M. Carol, P. Cano, L. Clotet, A. Despuig, L. Curto, J. Ferràs, G. Ferrús, R.M. Vileu, N. Follia, M. Sabater, E. Plasencia, M. Bosch, T. Pérez-Porcuna, À. Tarrés, M. López, H. Martínez. Public Health Agency of Barcelona: R. Prieto, J. P. Millet, C. Rius. Laboratories: S. Esteban-Cucó and E. Vicente (Laboratori de Referència de Catalunya, El Prat de Llobregat, Barcelona), G. Tudó (Universitat de Barcelona and Hospital Clínic-ISGlobal, Barcelona), J. González (Hospital Clínic-ISGlobal, Barcelona, and CIBER en Enfermedades Infecciosas (CIBERINFEC), Madrid), M. T. Tórtola (Hospital Universitari Vall d’Hebron, Barcelona and CIBERINFECT, Madrid), T. Soler and M. D. Guerrero (Consorci del Laboratori Intercountyl (CLILAB) de l’Alt Penedès, l’Anoia i el Garraf, Vilafranca del Penedès), I. Prats (Hospital Universitari Arnau de Vilanova, Lleida), F. Alcaide and L. Fernández (Hospital Universitari de Bellvitge-IDIBELL, L’Hospitalet de Llobregat), E. Cuchí (CATLAB-Centre Analítiques Terrassa AIE, Terrassa), M. Garrigó (Hospital de la Santa Creu i Sant Pau, Barcelona), P. Costa (Laboratori Clínic Territorial de Girona, Hospital Universitari de Girona Dr. Josep Trueta, Girona), A. Casabella (Àrea de Microbiologia, Servei de Laboratoris Clínics, Parc Taulí Hospital Universitari, Institut d’Investigació i Innovació Parc Taulí (I3PT-CERCA), Sabadell), Á. Pulido (Hospital General de Granollers, Granollers), E. Picó-Plana (Hospital Universitari Joan XXIII, Tarragona), J. López and G. Trujillo (Fundació Althaia, Hospital Sant Joan de Déu, Manresa), N. Torrellas (Laboratori Fundació Hospital de Palamós, Palamós), X. Casas (Serveis Clínics, Barcelona). Universitat de Barcelona, CIBERESP: P. Godoy and Á. Domínguez. Centre d’Estudis Epidemiològics del VIH/SIDA de Catalunya, CIBERESP: E. Muntada, E. López-Corbeto, and J. Casabona.
