# Supplementary material for: Population structure and spatial distribution of Mycobacterium tuberculosis complex in Catalonia
Source: Front Microbiol. 2026 Mar 20;17:1787894. doi: 10.3389/fmicb.2026.1787894 (PMC13047082; doi:10.3389/fmicb.2026.1787894)
Supplement: Supplementary file 3 [file Supplementary_file_1.docx]

Supplementary Material


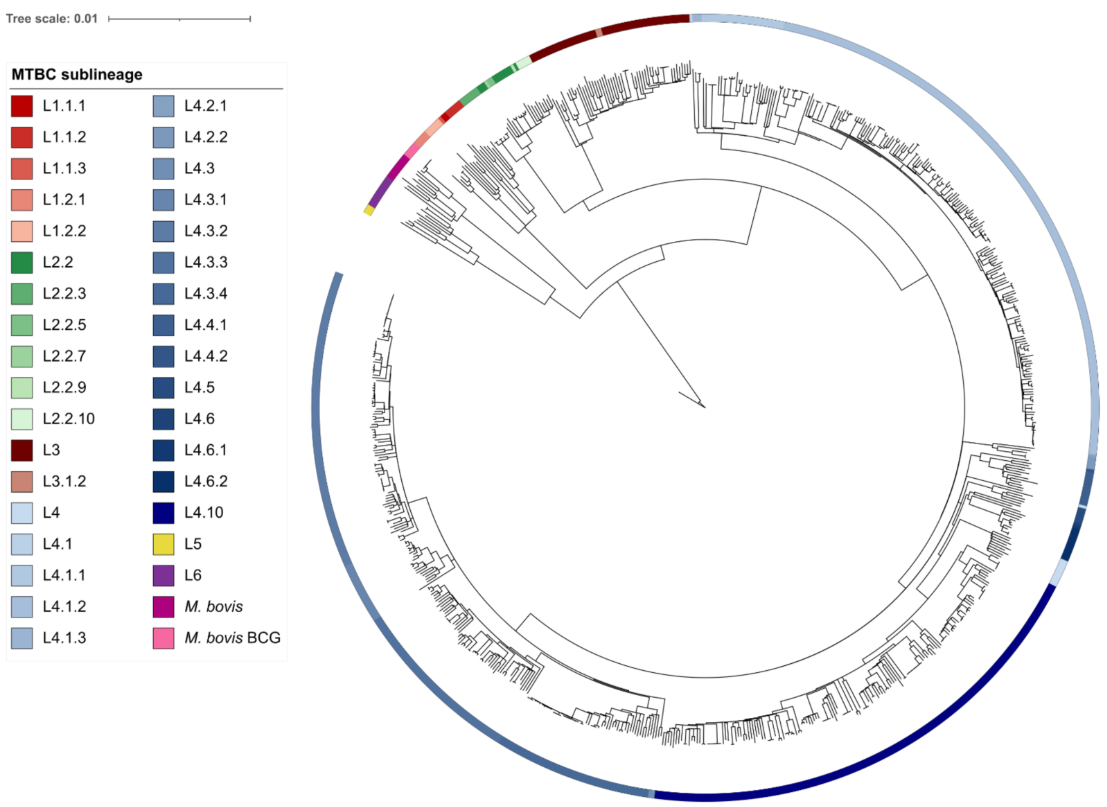


**Supplementary Figure 1.** Maximum likelihood phylogenetic tree of genomic sequences of 783 MTBC isolates in Catalonia
