# Supplementary material for: Population structure and spatial distribution of Mycobacterium tuberculosis complex in Catalonia
Source: Front Microbiol. 2026 Mar 20;17:1787894. doi: 10.3389/fmicb.2026.1787894 (PMC13047082; doi:10.3389/fmicb.2026.1787894)
Supplement: Supplementary file 4 [file Supplementary_file_2.docx]

Supplementary Material


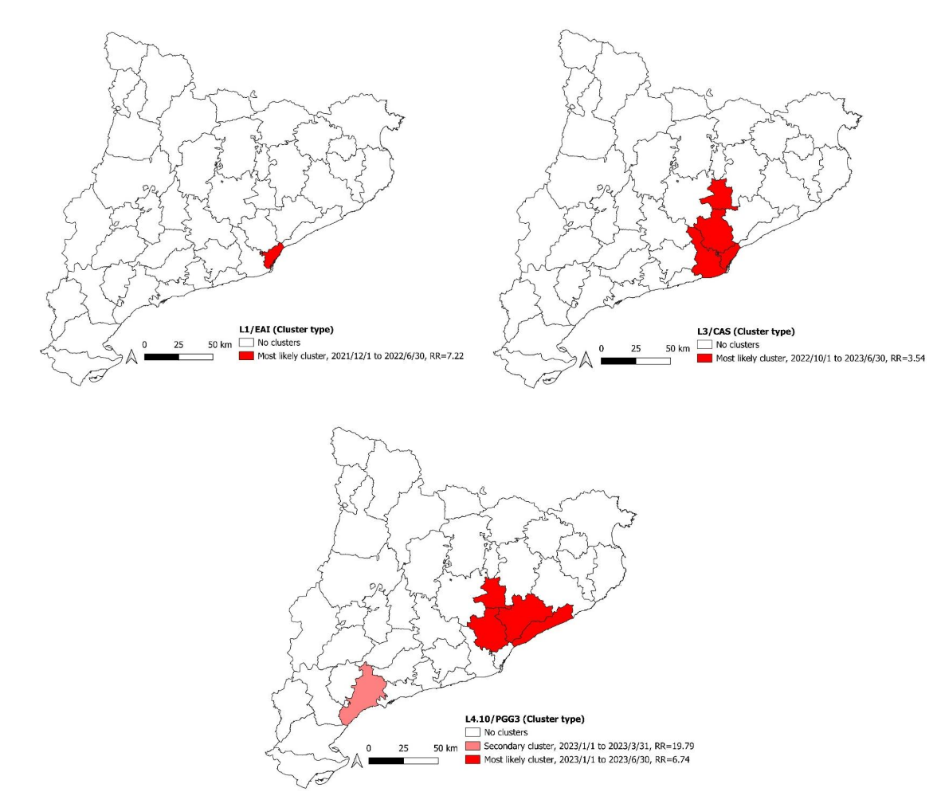


**Supplementary Figure S2**. Spatiotemporal clustering of MTBC (sub)lineages. The cluster map shows the distribution of hotspot areas for MTBC (sub)lineages in Catalonia, December 2021-June 2023 (1.5 years). Non-significant clusters are not shown
